# Assessment of exocrine pancreatic function in children and adolescents with direct and indirect testing

**DOI:** 10.3389/fped.2022.908542

**Published:** 2022-11-14

**Authors:** Puanani Hopson, Yamen Smadi, Vijay Mehta, Samit Patel, Devendra Mehta, Karoly Horvath

**Affiliations:** ^1^ Department of Children Center, Pediatric and Adolescent Medicine, Gastroenterology and Hepatology, Mayo Clinic, Rochester, MN, United States; ^2^ Center for Digestive Health and Nutrition, Arnold Palmer Hospital for Children, Orlando, FL, United States; ^3^ Pediatric Gastroenterology & Nutrition of Tampa Bay, Tampa Bay, FL, United States

**Keywords:** pancreas, exocrine function, acinar cells, duct cells, indirect pancreatic function test, direct pancreatic function test

## Abstract

The exocrine pancreas plays an important role in digestion. Understanding of the physiology and regulation of exocrine function provides insight into disease processes and basis of functional testing. Specifically, exocrine pancreatic insufficiency (EPI) can cause maldigestion and thus a proper assessment of exocrine pancreatic function is important. There are indirect and direct methods for evaluating pancreatic function. Indirect methods are varied and include stool, serum, urine, and breath tests. Fecal elastase is a commonly used indirect test today. Direct methods involve stimulated release of pancreatic fluid that is collected from the duodenum and analyzed for enzyme activity. The most used direct test today is the endoscopic pancreatic function test. Indirect pancreatic function testing is limited in identifying cases of mild to moderate EPI, and as such in these cases, direct testing has higher sensitivity and specificity in diagnosing EPI. This review provides a comprehensive guide to indirect and direct pancreatic function tests as well as an in-depth look at exocrine pancreatic function including anatomy, physiology, and regulatory mechanisms.

## Introduction

The exocrine pancreas plays a crucial role in digestion and as such, its function is crucial in pediatric population where growth and development are reliant upon adequate nutrition. The objective of this article is to provide a comprehensive review of the exocrine pancreas and discuss options to evaluate its function.

## Anatomy of the pancreas

The pancreas consists of 5 different parts, the head, uncinate process, neck, body, and tail. The head and uncinate process located near the portal vein, superior mesenteric vein, and superior mesenteric artery. This may be a possible explanation why severe acute pancreatitis can be seen with systemic inflammatory reactions.

The pancreas has both exocrine and endocrine functions. The exocrine pancreas encompasses roughly 85% of the pancreatic mass where 10% of the gland is accounted for by extracellular matrix, 4% by blood vessels and the major ducts, and only 2% of the gland is comprised of endocrine tissue (1). The exocrine and endocrine functions are coordinated to allow a regulatory feedback system for digestive enzyme and hormone secretion. Specifically, the blood flow from the endocrine pancreas enters the capillaries of the exocrine tissue before entering the general circulation, and in the exocrine tissue, there are insulin receptors that are involved in regulation of digestive enzyme synthesis (2–4).

The exocrine pancreas is composed of **acinus** (a collection of about 40 acinar cells) and its **draining ducts** (5). The centro-acinar cell functions as an extension of the ductal epithelium into the acinus and provides progenitor cells important for pancreatic regeneration (6, 7). The **acinar cells** synthesize digestive enzymes (lipases, amylase, and proteases) to be stored in zymogen granules and then secreted (enzyme-containing zymogen granules fuse with the apical cell membrane surface) (8). The ductules drain into interlobular (intercalated) ducts and then into the main pancreatic ductal system.

Thanks to its highly developed endoplasmic reticulum (ER) system, the acinar cell of the exocrine pancreas has one of the highest protein synthesis rates among mammalian organ (9, 10). The ER is also a major storage site for intracellular calcium, which, when released into the cytoplasm, is a mediator for secretion of stored digestive enzymes into the pancreatic ductal system (11).

Another cell type known for its role in pathologic states is the **pancreatic stellate cell** that has a role in pancreatic fibrosis (12, 13). They are found around the acinar and ductular structures as well as the islets of Langerhans. In chronic pancreatitis the stellate cell is transformed into a proliferating myofibroblast cell type that synthesizes and secretes extracellular matrix proteins, proinflammatory cytokines and growth factors (14).

## Physiology and regulation of pancreatic secretion

The adult pancreas secretes up to 2500 ml of colorless, odorless, alkaline, isosmotic pancreatic fluid. The flow and concentration of this fluid is highly regulated. The flow rate increases from an average rate of 0.2 or 0.3 ml/min in the resting (interdigestive) state to 4.0 ml/min during postprandial stimulation (15). The ratio of the different enzymes released is adjusted to the composition of digested food. For example, a carbohydrate-rich diet results in an increase in synthesis of amylase and a decrease in chymotrypsinogen (16), while a lipid-rich diet enhances lipase synthesis (17).

### Electrolyte secretion

The principal compounds secreted by the exocrine pancreas are water, sodium, potassium, chloride, and bicarbonate. The osmolality of pancreatic juice is independent of flow rate.

Secretin is the main stimulant of electrolyte secretion from ductal and centroacinar cells. Secretin was the first hormone ever discovered at the beginning of 20th century (18). Secretin is released from enteroendocrine S cells in the duodenal mucosa when the pH of the lumen is less than 4.5 (19). Binding of secretin to its receptor activates adenylate cyclase, resulting in the generation of cyclic adenosine monophosphate (cAMP), which acts as the intracellular messenger. The duct cells and centroacinar cells contain carbonic anhydrase, which is important for their ability to secrete bicarbonate (20).

Presence of bicarbonate secretion in the proximal pancreatic ducts is largely mediated by a chloride and bicarbonate exchange transporter. In distal ducts, the luminal bicarbonate concentration is already high, and thus the bicarbonate secretion is mediated by bicarbonate conductance *via* the cystic fibrosis transmembrane conductance regulator (CFTR) (21). The secreted bicarbonate acts to buffer the acidic fluid entering the duodenum from the stomach and brings this fluid pH to the optimal level for pancreatic enzyme function.

The concentration of bicarbonate secreted can vary based upon the secretory rate of the pancreas. In resting state, the chloride concentration is high in the pancreatic fluid. Alternatively in an active state following secretin stimulation, the bicarbonate concentration is significantly increased. Bicarbonate concentration thus serves as a great marker for pancreatic function, and in testing discussed in detail later, a bicarbonate level lower than 80 mEq/L it is considered abnormal (22, 23).

### Pancreatic enzyme secretion

The acinar cells release pancreatic enzymes from their zymogen granules into the lumen of the acinus, and these proteins combine with the water and bicarbonate secretions of the centroacinar and duct cells.

The exocrine secretion has significant reserve capacity. DiMagno et al. (24) investigated this by plotting lipase output and fecal fat excretion in patients with EPI. They reported that fecal fat excretion was increased when the lipase output fell below 10%. Later they found that maldigestion and malabsorption do not occur until the digestive enzyme secretion (when stimulated by CCK) is reduced to 5% to 10% of normal values (25).

### Stimulation of pancreatic enzyme secretion

Pancreatic enzyme secretion is stimulated both by neural and humoral mechanisms.

#### Neural mechanisms

Direct vagal and regional reflexes stimulate pancreatic enzyme secretion. The vagal stimulation activates the cholinergic, muscarinic receptors (M3) with resultant generation of intracellular cyclic guanosine monophosphase (cGMP). The vagus-mediated cephalic phase of pancreatic secretion in humans and experimental animals results in pancreatic fluid that is low-volume with high enzyme concentration.

Distention at the gastric antrum elicits pancreatic enzyme secretion by activation of a vago-vagal reflex called the antro-pancreatic reflex (26). The antro-pancreatic reflex is an important component of the gastric phase of pancreatic secretion (27).

#### Humoral mechanisms

Cholecystokinin (CCK) is the major humoral mediator of enzyme secretion during the intestinal phase. Specifically, the presence of fat and protein products in the intestine will trigger release of CCK-releasing peptide that then act on CCK containing cells (I-cells) to release CCK (28).

In addition to CCK, other peptide hormones (e.g., secretin, neurotensin) and neurocrine agents (e.g., GRP, PACAP) can stimulate enzyme secretion (29). However, as mentioned above, secretin has cental role in stimulating electrolyte and bicarbonate secretion.

The effect of CCK is mediated *via* a specific receptor (CCK-A receptor) that can be found on acinar cells, intrapancreatic neurons, and cholinergic afferent neurons. In humans, pancreatic enzyme secretion in response to CCK stimulation or food is inhibited by atropine and somatostatin (30–32). This suggest that CCK's action on the pancreas is dependent on cholinergic mechanism.

Several other peptides including PACAP, GRP, and neurotensin can also act to stimulate pancreatic enzyme secretion (29). However, the extent to which these peptides play a role pancreatic enzyme secretion in humans is not well known.

### Enzyme secretion products

#### Amylase

Pancreatic **amylase is secreted in its active form.** Amylase acts to break down starch and glycogen to glucose, maltose, maltotriose, and dextrins. The 2–9 glucose units are further breaking down by the small intestinal brush border enzymes. These simple sugars are then absorbed *via* the active transport mechanisms along the brush border of the intestinal epithelial cells.

#### Proteases

Proteins are first hydrolyzed into peptides in the stomach. These peptides then go on to the intestine and stimulate release of CCK-releasing peptide, CCK, and secretin, which then stimulate the pancreas to secrete enzymes and bicarbonate into the intestine.

The proteolytic enzymes include **trypsinogen, elastase,** and **carboxypeptidase A and B**. They are **secreted as proenzymes** that require activation. Trypsinogen is converted to its active form trypsin, by another enzyme, **enterokinase**, which is produced by the duodenal mucosal cells (33). Trypsin, in turn, activates the other proteolytic enzymes. Together, these enzymes cleave bonds between amino acids, so that they can be actively transported into the intestinal epithelial cells for absorption.

To prevent activation of these enzymes while in the pancreas, the acinar cells produce a trypsinogen inhibitor. A failure to express this trypsinogen inhibitor, pancreatic secretory trypsin inhibitor (PSTI), also known as serine protease inhibitor Kazal type 1 (SPINK1), is a known cause of familial pancreatitis.

#### Pancreatic lipases

The **pancreatic lipase** acts to break down triglycerides. Unlike the proteases discussed above, lipase is secreted in an active form. **Colipase** is also secreted by the pancreas and acts to enhance activity of lipase by binding to it and changing its molecular configuration.

**Phospholipase A2** is secreted by the pancreas as a proenzyme and requires activation by trypsin. Phospholipase A2 hydrolyzes phospholipids.

**Carboxylic ester hydrolase and cholesterol esterase** act to break down lipid substrates, such as esters of cholesterol, fat-soluble vitamins, and triglycerides. These can then be then packaged into micelles for transport into the intestinal epithelial cells.

The diminished or absent lipase secretion leads to steatorrhea, one of the main clinical symptoms of exocrine pancreatic insufficiency. In our diet, fats are mainly long-chain triglycerides that are broken down into two fatty acids and one beta monoglyceride by the pancreatic lipase.

Pancreatic lipase is degraded when the luminal pH drops <4, therefore diseases that result in acidic intraluminal environments (pancreatic duct cell dysfunction, excessive gastric acid section, etc.) can inhibit fat digestion. This is the main reason that pancreatic enzyme replacement preparations have enteric coated granules.

Gastric lipase is a non-pancreatic lipase that acts to hydrolyze fats; however, it cannot fully compensate for the absence of pancreatic lipase. Infants rely upon other enzymes secreted from the pancreas (pancreatic triglyceride lipase (PTL)-related protein 2, and bile salt-stimulated lipase (BSSL)) that act in conjunction with gastric lipase to achieve efficient fat absorption (34). Interestingly, BSSL is also present in human milk, which facilitates fat absorption and growth in breast-fed infants. Table 1 lists the enzymes and their substrates and products.

**Table 1:** Summary of the enzymes produced by the acinar cells and their actions.

Enzyme	Substrate	Products
**Carbohydrate digestion**		
Amylase (active)	Starch, glycogen	Glucose, maltose, maltotriose, dextrins
**Protein digestion**		
**Endopeptidases**		
Trypsinogen (inactive)	Cleave bonds between amino acids in proteins	Amino acids, dipeptides
Chymotrypsinogen (inactive)		
Proelastase (inactive)		
**Exopeptidases**		
Procarboxypeptidase A&B (inactive)	Cleave amino acids from the end of the peptides	Amino acids
Carboxypeptidase A&B (active)		
**Fat digestion**		
Lipase (active)	Triglycerides	Fatty acids, Β-monoglycerides
Phospholipase A2 (inactive)	Phospholipids	
Cholesterol esterase	Neutral lipids	

### Inhibition of pancreatic secretion

Inhibition of exocrine pancreatic secretion occurs through several mechanisms. Somatostatin, pancreatic polypeptide (PP), peptide YY (PYY), neuropeptide Y pancreastatin, and glucagon are all peptides that inhibit secretion indirectly through the activation of inhibitory intrapancreatic neurons. Somatostatin is produced by the delta cells in islets of Langerhans and it exerts an inhibitory effect on amino acid uptake as well as enzyme and bicarbonate secretion (32, 35).

#### Feedback regulation

Feedback regulation was studied in human and animals by first noting that when pancreatic fluid was diverted from the intestine an increase in pancreatic fluid secretion occurred (36). This augmented enzyme secretion occurred secondary to a rise in circulating CCK (37).

Alternatively, the increase in CCK and pancreatic fluid secretion into the intestine is inhibited by presence of trypsin in the intestine as well as other digestive enzymes (38). This feedback is accomplished *via* the CCK-releasing peptide in such that with the absence of peptides, CCK-releasing factor will be inactivated by trypsin and thus CCK secretion is decreased (28).

### Phases of exocrine secretion

#### Interdigestive secretion

There is fluid secretion in the fasting (interdigestive) stage that is cyclic and follows the pattern of the migrating myoelectric complex (MMC) (39, 40). This pattern occurs every 60 to 120 min, with bursts of enzyme and bicarbonate secretions being released. Also, there is bile secreted from the gallbladder following partial gallbladder contraction during phases of the MMC. This provides a housekeeping function by cleaning the debris from the small intestine. This process involves the cholinergic nervous system and the hormones motilin and pancreatic polypeptide (39, 40).

#### Digestive secretion

##### Cephalic phase

Cephalic phase is mediated by the vagus nerve. In humans, the cephalic phase was identified in studies utilizing a sham feeding method by which the participant would chew food and spit it out. One study (41) indicated that this sham feeding stimulated pancreatic enzyme secretion that rose to about 90% at its maximum, and bicarbonate was also secreted. Atropine suppressed basal trypsin output and essentially abolished the response to sham feeding (42). This suggests that acetylcholine is a major neurotransmitter involved in mediating cephalic phase of pancreatic secretion (39). Among the hormones, gastrin-releasing peptide (GRP) is released from the pancreas upon vagal stimulation and may mediate enzyme secretion (43).

##### Gastric phase

Gastric phase is initiated by gastric distention by meals. This phase results in secretion of pancreatic enzymes with little effect on the secretion of water and bicarbonate. In studies mimicking gastric distention (fundus or antrum) with a balloon, a resultant low-volume enzyme-rich secretion was obtained through a gastropancreatic vago-vagal reflex (44). Output of gastric contents into the duodenum (gastric chyme with peptides and fatty acids) also act as stimulus at the level of the intestinal mucosa and begins the intestinal phase of pancreatic secretion through neural and hormonal mechanisms. Thus, the rate of gastric emptying can play an important role in pancreatic secretion. As such surgery that alters emptying can often lead to augmented signaling and mixing of gastric and pancreatic fluids.

##### Intestinal phase

Intestinal phase is mediated by entero-pancreatic vago-vagal reflexes and various hormones. This phase starts when chyme enters the small intestine from the stomach. Specifically, the chyme consists of hydrogen ions, fatty acids, amino acids, and peptides, and these have roles in the intestinal phase of pancreatic section (45). Of the amino acids, phenylalanine, valine, methionine, and tryptophan are known to cause a more robust pancreatic secretory response (46).

Ductal secretion is initiated by hydrogen ions, creating a low pH environment (pH below 4.5) that triggers secretin release from enteroendocrine S cells (19).

The magnitude of stimulation of the pancreas varies not only by the type of nutrients but also by the site of delivery of the nutrients (47). Elemental diet causes less pancreatic enzyme secretion compared to a standard meal, and delivery of nutrients to the jejunum causes less pancreatic secretion than delivery to the duodenum (47).

Vago-vagal reflexes were found to play a role in pancreatic enzyme and bicarbonate secretion. Particularly studies with vagotomy led to low intestinal loads of amino acids and fatty acids, and studies with atropine led to lower physiologic concentrations of CCK (48, 49).

## Assessment of exocrine function

Since the 1940s there have been many tests developed to assess the exocrine pancreatic function. They Include tests that can assess the function of a single enzyme from the stool, serum, urine, or by breath test (indirect tests) and ones that assess the activity of several digestive enzymes from stimulated pancreatic fluid (direct functional tests). The main indications of the exocrine function assessments are listed in Table 2.

**Table 2:** Indications for the assessment of exocrine pancreatic function.

**Signs or symptoms concerning for EPI**
Failure to thrive
Steatorrhea
Chronic diarrhea
Chronic abdominal pain
Abdominal bloating
Borborygmi
Fat soluble vitamin deficiencies
**Genetic syndromes associated with EPI**
Cystic fibrosis
Shwachman-Diamond syndrome
Johanson Blizzard syndrome
Pearson syndrome
**Assess PERT**
Non-compliance with treatment
Measure PERT dose adjustment

PERT, pancreatic enzyme replacement therapy.

### Development of enzyme secretions

It is important to understand the intrauterine and postnatal development of enzyme secretion for the accurate interpretation of the functional test results.

Intrauterine development of amylase, lipase, and trypsinogen secretion does not occur at the same time (50, 51). Trypsinogen and chymotrypsinogen were found to be present around 14 to 16 weeks, followed by lipase first appearing by 21 weeks of gestation. Lipase is uniformly present by postnatal age of 15 days (50). Amylase detection is postnatally and occurs much later than all other enzymes. Lebenthal and Lee et al. reported that infants at 30 days old have no detectable amylase activity in duodenal fluid; however, children at around 2 years of age had normal adult level of amylase activity (52, 53).

This postnatal appearance of amylase and lipase in infants may not cause symptoms in breast-fed infants as breast milk has significant amylase (54) and bile salt–dependent lipase in breast milk (contributes to lipid digestion in infants) (55).

There are case reports of isolated lipase/colipase deficiency, detected by duodenal fluid aspiration in children with clinical presentation of greasy stools (56–62). Additionally, isolated amylase deficiency was identified in a large retrospective pediatric database of endoscopic pancreatic function testing (ePFT) (63). An error in mRNA processing or protein secretion was suggested by Mehta et al. in a reported pediatric case with isolated amylase deficiency diagnosed after repeated ePFTs (20 and 33 months of age), despite detecting normal pancreatic amylase messenger RNA by reverse-transcriptase polymerase chain reaction in the duodenal fluid (64). Understanding of isolated pancreatic enzyme deficiencies as pathologic or physiologic is overall limited and represents area for future research.

## Indirect exocrine function tests

Indirect function tests are based upon the function of a single enzyme. They measure individual pancreatic enzymes or their substrate byproducts from stool, serum, or breath samples. The examples of these tests are fecal fat, steatocrit, fecal elastase (FE-1), stool chymotrypsin, serum markers, and the ^13^C-mixed triglyceride breath test. Each indirect test has its own inherent limitations; however, they all share a common limitation of poor sensitivity and specificity in detecting mild to moderate EPI.

### Stool based tests

#### Fecal elastase test

Fecal elastase (FE-1) is the most widely used indirect screening test for EPI. The basis of this tests that the elastase is resistant to hydrolysis by bacterial proteases and it remains stable in room temperature (65). A small stool sample is adequate for the test. The other advantage is that pancreatic enzyme replacement therapy (PERT) does not interfere with the result. Therefore, discontinuation of PERT is not necessary when performing the FE-1 test (66).

FE-1 has been well studied in pancreatic exocrine dysfunction associated with chronic pancreatitis, cystic fibrosis, diabetes, and celiac disease (67–70). The normal result is >200 mg/g of dry stool. A level <200 mg/g indicates EPI, and <100 mg/g correlates well with steatorrhea (71). Khan et al. proposed a method of staging EPI (into mild, moderate, and severe) based upon value of FE-1 combined with presence of symptoms and fat soluble vitamin deficiency (72).

It is important to note that large volume liquid stool can dilute the fecal elastase and provide inaccurate results, therefore for the correct analysis the stool sample should be lyophilized, and dry weight should be uses for calculations (73).

Diet is not suggested to have an large impact on FE-1 testing, however Walkowiak et al. reported that in pancreatic sufficient patients with normal range FE-1, a short term vegan diet did lower their FE-1 suggesting possible adaptation of pancreatic proteases to low protein high fiber diet (74).

The sensitivity of the FE-1 in children with CF is between 86% and 100% (71, 75, 76). In a meta-analysis, FE-1 of <200 mgc/g was found to have an overall pooled sensitivity of 77% and specificity of 88% in detecting EPI (77). As expected, the accuracy of FE-1 increases in cases of severe EPI (sensitivity of 97%) and alternatively decreases in cases of isolated deficiency or mild EPI (sensitivity 49%).

Although the FE-1 can only detect EPI reliably in the severe range, it remains more sensitive than fecal fat testing. Isolated enzyme deficiencies are not detected by FE-1, for example, steatorrhea secondary to isolated lipase or colipase deficiency (78).

#### Stool fat content measurement

Assessment of fecal fat is a standard method to detect fat malabsorption. The causes of fat malabsorption are varied, and as such, a positive test is neither specific nor sensitive to exocrine pancreatic dysfunction. It is an indirect assessment of lipase activities of the pancreas. This test measures the fraction of fat in the stool after initiating a standard fat containing diet. However, this procedure is not specific for lipase activity as there are other non-pancreatic etiologies of the abnormal fecal fat detection. These include gut mucosal injury (e.g., celiac disease), small bowel bacterial overgrowth, short bowel syndrome, Crohn disease, and even liver disease with cholestasis (79, 80). In patients with cystic fibrosis (CF) without pancreatic insufficiency, fat malabsorption can occur due to gastric hypersecretion or an abnormal gastrointestinal motility (81). In a study in patients with Shwachman-Diamond syndrome (SDS) and CF, steatorrhea developed when lipase fell below 2% or colipase fell below 1% (82) and as such that are clear cases here fecal fat testing would likely be positive.

Fecal fat testing is a cumbersome test for the patient and laboratory to perform; thus, has fallen out of favor as first line testing in many clinical settings. The test includes all stool collection for 72 h while the total fat intake (100 g/day) is standardized starting 3 days before and during the full 3 days of collection (83). Then the ratio of stool fat content compared to total fat intake is calculated. Typically, a level of >7 g/day is defined as malabsorption (79). It is a time-consuming test, although there is a report that 24-hour collections are adequate (84). It is important to store the collected fat in a refrigerator, otherwise the bacteria in stool will start fermenting the fat and fat content may decrease.

It is well known that the fat absorption ratio is age dependent. In children that are younger than 6 months of age, the reference values are >85%, and above that age, reference values are >93% to 95% (85, 86).

The classical method for stool fat analysis was quantitative testing *via* the Van de Kamer method. However, the near-infrared reflectance analysis simplified the quantification, and this correlates well with the classical Van de Kamer method (87, 88)*.* The qualitative stool fat test is based on the use of Sudan stain of the stool and microscopic analysis of fat droplets and results are reported in a graded fashion (1 + normal; 2 + slight increase; and 3 + definite increase) (89). Qualitative analysis is lacking in its ability to separate normal from mild or inconsequential cases of steatorrhea.

The coefficient of fat absorption is another measure obtained with fecal fat testing. A value <90% is defined as insufficient, the calculation is: (fat ingestion − fat excretion)/fat ingestion) × 100(%). However, Erchinger et al. reported that for the diagnosis of fat malabsorption, the additional evaluation to calculate the ratio of fat absorption did not provide additional information compared to fecal fat content (90).

#### Steatocrit

Steatocrit is a fast and easily performed screening test for fat malabsorption. Recall that fat malabsorption has pancreatic and non-pancreatic etiologies, thus a positive steatocrit is not specific to pancreatic insufficiency. This test includes the collection of stools that is then homogenized and an aliquot of sample transferred to hematocrit tube and centrifuged at 12,000 rpm for 15 min. The ratio of the fat layer to the total sample length is assessed. After the test introduction in infants in 1981 (91) this simple, cheap and rapid test became popular. However, it has poor sensitivity and specificity compared with the 72-hour stool fat collection. Tran et al. reported that the sensitivity of the test can be improved *via* acidification of the stool sample prior to centrifugation (acid steatocrit test) (92, 93).

#### Stool chymotrypsin

Chymotrypsin in stool is detected by a photometric assay test (93). Unlike elastase, chymotrypsin is prone to proteolytic degradation and can limit the availability and handling of the test. Another limitation is that the test cannot differentiate human chymotrypsin from the chymotrypsin found in PERT (94). Thus, PERT must be stopped at least 3 days before the test. When compared to ^13^C mixed triglyceride breath testing and FE-1, fecal chymotrypsin had the lowest sensitivity and specificity at 56% and 82%, respectively (95). The test's main advantage can be that it allows assessing compliance to PERT.

### Urine based test

#### Pancreolauryl test

The substrate for the pancreaolauryl test is dilaurate (lauric acid, a 12-carbon atom chain fatty acid, and a component of triglycerides that comprises about half of the fatty-acid content in coconut milk) combined with fluorescein. Pancreatic lipase releases the fluorescein that is then absorbed and can be measured in the urine (96) and blood (97). Later the test was modified by adding mannitol to correct for changes in intestinal permeability that could affect absorption and skew the test results (98). The results are reported as a fluorescein/mannitol ratio. However, when compared with FE-1 test, the pancreolauryl test was less accurate (99).

### Serum tests

Serum testing for EPI has fallen out of favor for reasons discussed below, however understanding of these tests in relation to other pancreatic diseases and in monitoring secondary effects of EPI are important.

Amylase and lipase, are present in the blood stream due in part to physiologic release or leaking of these from the acinar cells into the systemic circulation. Thus, pancreatic disease states with inflammation can lead to elevation of these. Alternatively, atrophy or significant loss of pancreatic tissue can cause a decrease in amylase and lipase.

In the 1980s, serum IRT was found to have sensitivity and specificity in diagnosing severe cases of EPI, in which a result of less than 20 ng/ml was consistent with pancreatic steatorrhea, compared with levels higher than 20 ng/ml in those without steatorrhea (100). Interestingly around that time, IRT was recognized in dried blood spots in neonates found to have cystic fibrosis and was later adopted into the newborn screening. Adoption of serum IRT for EPI fell out of favor due to the significant limitations in age reported by Durie et al. (101) and the advent of other more specific pancreatic function tests. Thus, outside of neonatal screening, IRT is no longer used clinically for assessment of exocrine pancreatic function.

Other serum tests associated with downstream effects of EPI include decreased serum levels of fat-soluble vitamins, apolipoproteins, total cholesterol, magnesium, retinol-binding protein, calcium, zinc, selenium, and carotene (102). It was reported that patients with EPI are at risk for vitamin E deficiency (103, 104), that can lead to neurological symptoms, highlighting the importance of these adjunctive serum tests in detecting complications in EPI*.* Additional tests may include hemoglobin, albumin, prealbumin, and HbA1c, as well as diminished bone density, all of which can be abnormal in the setting of untreated EPI (105).

### Breath test

#### ^13^C mixed triglyceride breath test

The ^13^C is a natural nonradioactive form of the carbon. The test measures ^13^C–CO_2_, which is one of the breakdown products of digested triglycerides (106). This test is based on the function of lipase, however, like the fecal fat assay, the ^13^C-mixed triglyceride breath test is a test of fat maldigestion and is not specific to EPI.

The ^13^C mixed triglyceride breath test was first described by Vantrappen et al. in 1989 (107). The test utilizes a ^13^C-labelled mixed triglyceride [1,3-distearyl,2 (carboxyl-13C) octanoyl glycerol] substrate that is consumed with a meal, typically butter (or similar fat) on toast. This fat is then hydrolyzed by the pancreatic lipase (and/or other non-pancreatic fat digestion processes) and the ^13^C-labelled octanoate, an 8-carbon medium-chain fatty acid, is absorbed in the blood and metabolized by the liver and the ^13^C-labelled CO_2_ appears in the expired air of the patient. The^13^CO_2_ is detected in breath samples at various time points throughout a 5–6-hour study. The result of the test is expressed as percentage of ^13^C cumulative recovery over the testing period, with values in normal subjects being between 20%–40% of cumulative recovery (106). The ^13^CO_2_ is measured by mass spectrometry or near-infrared analysis.

The amount of ^13^C-labelled CO_2_ is an indirect measure of pancreatic lipase activity, although as mentioned above, there may be other non-pancreatic diseases influencing the result. The main advantage of the ^13^C-mixed triglyceride breath test is in its ability to assess the efficacy of PERT. The limitations of the test are that there is a wide variability in the amount of expired ^13^C-labelled CO_2_, and these values can fluctuate with activity level, gastric emptying rate, liver disease, intestinal diseases that affect absorption, lung disease, and endogenous CO_2_ production (108–110). The breath test is also difficult to perform in infants and young children.

The 13C-mixed triglyceride breath test is widely published (111–114), however, currently it is only available in a few countries in Europe and in Australia.

## Direct (stimulatory) exocrine function tests

Direct pancreatic function tests measure enzyme activity in pancreatic secretions. They are stimulated tests with either secretagogues (Secretin/CCK) or meal (Lundh test). They allow to assess the activity all the main pancreatic enzymes and provide option for other analyses of the collected fluids.

Direct pancreatic function test with secretagogue (secretin, cholecystokinin [CCK] administration is considered the gold standard to assess exocrine pancreatic function. In 1948, the first direct pancreatic function test was published (115). It used a specific double lumen tube to collect fluid samples from the duodenum (Dreiling tube) following simulation with secretagogue. Later a meal-based stimulation “Lundh meal test” was developed. This was then followed by the development of the endoscopic stimulation test in the 20th century. The advantages and disadvantages and clinical utility of the different tests are summarized in Table 3.

**Table 3:** Summary of the advantages, disadvantages, and clinical value of the different tests.

Test	Description	Advantages	Disadvantages	Clinical indications
**Direct (stimulatory) tests to assess all enzymes**				
Secretin	Measurements of resting duodenal enzyme activity in the first 10 min and bicarbonate secretion 15–60 min after IV secretin	Provide the most sensitive and specific measurements of exocrine pancreatic function (ePFT and Dreiling tube methods)	Require duodenal intubation and intravenous administration of hormones; not widely available	Detection of mild, moderate, or severe exocrine pancreatic dysfunction
Cholecystokinin	Measurements of duodenal outputs of amylase, trypsin, chymotrypsin. and lipase after IV administration			
Secretin and cholecystokinin	Measurements of volume, bicarbonate and enzymes activities after IV secretin and cholecystokinin}			
**Meal-stimulated test**				
Lundh test meal	Measurement of duodenal enzyme activities after oral ingestion of a test meal	Does not require IV administration of hormones	Requires duodenal intubation, a test meal, and normal anatomy, including small intestinal mucosa; not widely available	Detection of moderate or severe exocrine pancreatic dysfunction when a direct test cannot be done (i.e., limited availability of test)
**Indirect (non-stimulated) tests to assess a single enzyme function**				
Fecal fat	Measurement of fat in the stool after ingesting meals with a known amount of fat	Provides a quantitative measurement of steatorrhea	Requires sufficient dietary fat intake and collection of stool; only detects severe pancreatic dysfunction	Detection of severe exocrine pancreatic dysfunction and steatorrhea
Fecal chymotrypsin and Fecal elastase 1	Measurement of chymotrypsin or elastase 1 in the stool	Do not require IVs, tubes, or administration of oral substrates	Insensitive for detecting mild or moderate dysfunction	Detection of severe exocrine pancreatic dysfunction
Fluorescein dilaurate	Oral ingestion of fluorescein dilaurate with a meal, followed by measurements of fluorescein in urine or blood	Provide simple measurements for severe pancreatic dysfunction	Do not detect mild or moderate dysfunction; results may be abnormal in patients with small intestinal mucosal disease	Detection of severe exocrine pancreatic dysfunction
^13^C-Mixed Triglyceride Breath Test	It is consumed with a meal. Expired ^13^CO_2_ collected and measured by by mass spectrometry or near-infrared analysis	For the patients is an easy and convenient test	Requires special substrate and equipment and 5-6 h of breath collection. The result influenced by the intestinal function and liver metabolism	Detection of moderate and severe exocrine pancreatic dysfunction.

**Table 4:** Advantages and limitations of the ePFT.

Advantages of ePFT	Limitations of ePFT
• It allows assessing acinar and duct function combined with endoscopy. • It is significantly shorter than the traditional tube collection method. • Technically easy to perform and it is a safe procedure without patient's discomfort. • Enzyme measurements allow diagnosing isolated and generalized enzyme deficiencies in children. • It is helpful in the workup of malabsorptive diarrhea or poor weight gain.	• It can be performed only during anesthesia. • It slightly prolongs the duration of EGD assesses peak enzyme activities and not the total secretory capacity of pancreas. • Certain drugs used for anesthesia my influence the composition of the collected fluid.

EGD, esophago-gastro-duodenoscopy; ePFT, endoscopic pancreatic function test.

### Dreiling tube test

The Dreiling tube method (115) was considered a gold standard for the assessment of exocrine pancreatic function. Although the test is considered highly sensitive and specific (22, 116–122), the Dreiling tube collection method has inherent limitations.

The process of collection *via* the Dreiling tube starts with placement of an oro-duodenal tube (guided by fluoroscopy), baseline fluid is collected, then sequential administration of secretin and CCK and collection of the outcoming pancreatic fluid *via* aspiration of duodenal contents at varying time points. The volume of aspirate, pH, bicarbonate concentration, total protein concentration, and pancreatic enzyme activity are recorded. Amylase, trypsin, chymotrypsin, and lipase all can all be assayed and are reported as total enzyme output determined by the volume of fluid collected.

Multiple factors can influence the results of this test including mixing of gastric acid with intestinal fluid, inaccurate measure of “total volume” as the duodenal tube cannot reliably aspirate all secreted fluid, and dislocation of the tube (123).

The Dreiling tube collection method is invasive, impractical, difficult for patients to complete, and radiation exposure associated with verification of tube positioning, and can be time consuming to perform. Protocols for specimen collection in the publications are variable and the duration of the tests vary from 45 min to 150 min (124–127). In children specifically, this method of collection has never gained favor. Instead, many turn to non-invasive indirect testing such as fecal elastase.

### Lundh meal test

Another measurement of pancreatic function is the meal-based Lundh test (126). In this test, patients are asked to ingest a 300-mL liquid meal composed of dried milk, vegetable oil and dextrose (6% fat, 5% protein and 15% carbohydrate). This is then followed with the aspiration of fluid from the duodenum *via* a nasoduodenal tube, and measurement of enzyme activities. This is a physiological test that utilizes different phases of the meal (cephalic, gastric and intestinal), the effect of the meal on small intestinal sensory process, release of the secretin and CCK and the whole neurohumoral systems (vagal effects) and the pancreas responses to the neurohumoral system. Jensen et al. found significant correlation in lipase and bicarbonate concentrations between endoscopic secretin stimulation test and the Lundh test in 23 healthy volunteers (128).

### Endoscopic pancreatic function test (ePFT)

#### Method of fluid collection and analysis

**Figure 1:**
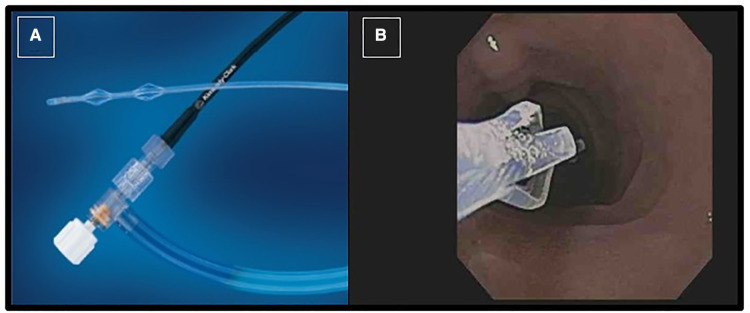
Picture of the collection catheter passed through endoscope working channel (**A**); the tip of catheter is seen in the duodenum close to the ampulla of vater (**B**).

The test is performed during a standard pediatric upper gastrointestinal endoscopy. Before endoscopic intubation, secretin or CCK is administered intravenously. For accurate collection of pancreatic fluid, the endoscope is positioned close to the ampulla of Vater and an aspiration catheter inserted through the biopsy channel (Figure 1) and with light suction is utilized. Pancreatic fluid secretion typically starts 3 to 4 min after the secretin administration, and the optimal collection time is within 10 min from the time of secretin injection.

There is a known dilutional effect of enzyme activities by ductal cell secretions if the fluid is collected beyond 10 min (129). Interpretation of the test results should be based on the sample with the highest (peak) enzyme activities (129, 130). However, the fluid secreted after 10 min reflects the effect of the secretin on the ductal cell function that is measured by bicarbonate concentration. In healthy subjects an increase in bicarbonate >80 mmol/L indicates normal function (123).

The fluid collected is measured for pH, protein content, and enzyme activity (amylase, lipase, trypsin, chymotrypsin, and elastase). The pH in the protein content of the fluid is utilized to assess the quality of the sample, in which a pH of less than 7 suggests possible contamination of gastric fluid and a low-protein would indicate dilution with duodenal fluid (131). Table 5 lists factors that can affect the result of the ePFT.

**Table 5:** Factors that have an effect on the results of ePFT (modified from (125).

	Factor	Effect
Substrate supply	Kwashiorkor and marasmus	Reduced stimulated enzyme output
	Protein repletion in kwashiorkor	Restored depressed pancreatic function
	Starch added to infant formula	Enhanced pancreatic *α*-amylase secretion
	Carbohydrate-rich diet in adults	No influence on amylase secretion
	Protein increase in infant formula	Augmented trypsin and lipase production
	Vegan diet	Decrease in the median of fecal elastase and chymotrypsin output
Celiac disease	Gluten in undiagnosed celiac patients	Temporary pancreatic dysfunction in 22.7% of newly diagnosed children
Ibd*	New or relapsed cases	Up to 40% had abnormal EPI
Drugs	Morphine	Increased bicarbonate and decreased protein secretion after 60 min
	Diazepam with hyoscine butylbromide	Reduced trypsin secretion
	Atropine	Decreased both basal and secretin-stimulated bicarbonate secretion
	Terbutaline	Inhibitory effect on the water and bicarbonate secretion
	Midazolam and meperidine	Did not affect the peak bicarbonate concentration or total bicarbonate output
Technique	Gastric acid contamination - low pH	Decreases pH below the pH optimum of enzymes and dilutes pancreatic fluid resulting in falsely low enzyme activity. Low pH also can denatures enzymes, especially lipase
	Collection of the fluid initially present in the duodenum	Duodenal secretions mixed with pancreatic fluid resulting falsely low enzyme activities
	Low protein	May result in unreliable enzyme assays
	Late collection	Due to increased water output the peak enzyme concentration per ml fluid can be falsely low (Figure 2)
	Bloody fluid	Mucosal injury resulting in blood contamination that influences the assays
	Single specimen	May result in low test sensitivity and specificity
Sample handling	Unfrozen specimen sent to the lab	It results in abnormally low enzyme activities

IBD, inflammatory bowel disease.

#### History and rationale of the ePFT

The first endoscopic fluid collection was reported in 1979 (130, 132). The first pediatric study comparing the Dreiling test with ePFT was reported by Madrazo et al. (133). Since then, multiple adult and a few pediatric papers (129, 133–135) have been published. In adults this test is most used to assess bicarbonate secretion that is an indicator of the ductal cell damage in chronic pancreatitis (CP). In contrast, in children the main role of this test is to determine the acinar cell enzyme secretion. The ePFT is more practical and efficient option for direct testing then the Dreiling and the Lundh tests to assess both the ductal and acinar cell functions.

The basis to assess acinar secretion is that secretin washes out the enzyme concentrated fluid that is present in the ducts prior to stimulation with secretagogue (interdigestive fluid). The interdigestive fluid in the pancreatic ducts has significant enzyme activity. This was found in several studies evaluating basal enzyme secretion was roughly 20% of the total pancreatic enzyme capacity, indicating that this basal secretion is adequate to prevent malabsorption and steatorrhea seen when pancreatic enzyme activity is <10% (136–138). Hence any dysfunction in enzyme secretion can be detected regardless of whether it is generalized insufficiency or an isolated enzyme deficiency.

#### Comparison of ePFT and dreiling tube method

The fluid collected in ePFT is analyzed and reported as peak enzyme activity in unit/ml/min (139). Alternatively, the Dreiling tube method reports the test results as total enzyme output by multiplying the enzyme activity and the volume of fluid collected.

The first pediatric study comparing the Dreiling tube and ePFT reported comparable results (133). Conwell et al. also compared the two collection methods in healthy adults and in patients with chronic pancreatitis using CCK infusion and reported that the ePFT was equivalent to the Dreiling tube collection (123). They also analyzed the safety and cost of the two tests which found that ePFT was safer, shorter in duration, and less costly ($1,890 vs. $2,659). The smaller fluid volume collected by ePFT reproduced the classic acinar and duct cell secretory profiles after hormonal stimulation (123, 137, 140). Based on these studies, ePFT was found to be a useful method for the assessment of pancreatic duct cell function (141, 142).

In conclusion, ePFT is comparable to the Dreiling tube method but offers several advantages over the Dreiling method. ePFT is less time consuming, does not result in patient's discomfort as performed during sedation, and eliminates need for radiation exposure. The limitations of ePFT include lack of uniformly accepted protocol, and requirement of anesthesia to perform (143). See list of advantages and limitations to ePFT in Table 4.

#### ePFT for acinar function in children

Following the first pediatric study in 1991 (133), Del Rosario et al. conducted a study in that one group of children received IV bolus of both CCK and secretin, while the second group received placebo after the administration of secretin and found no statistical difference in mean lipase level (129). The other important message of this study was that the peak enzyme values at the 5- and 10-minutes collections were similar in both groups, but the 15-minute specimens had significantly less enzyme activities due to dilution effect, and as such the optimal timing of collection was identified (129).

Another ePFT study in children, reported during the time of secretin shortage, compared secretin and CCK alone and in combination. It found that CCK was acceptable to be used alone for pancreatic enzyme measurements in the absence of commercially available secretin (135).

In 2016 a larger study with 508 ePFTs in children reported peak enzyme activities at 5 min that was then followed by a decrease activity over time (144). Additionally, they found discordance between ePFT and FE-1 testing in 165 children (144).

Up to now, the largest pediatric study included 1913 children and young adults summarized the experience with ePFT (secretin stimulated, collection time between 4 and 10 min) and determined that the test had high reproducibility, repeatability, and clinical validity (145). Additionally, by adding ePFT to standard upper gastrointestinal endoscopy when there was a suspicion of malabsorption, the diagnostic yield increased by 36.9% (145).

#### ePFT for ductal function test in children (single center data)

**Figure 2:**
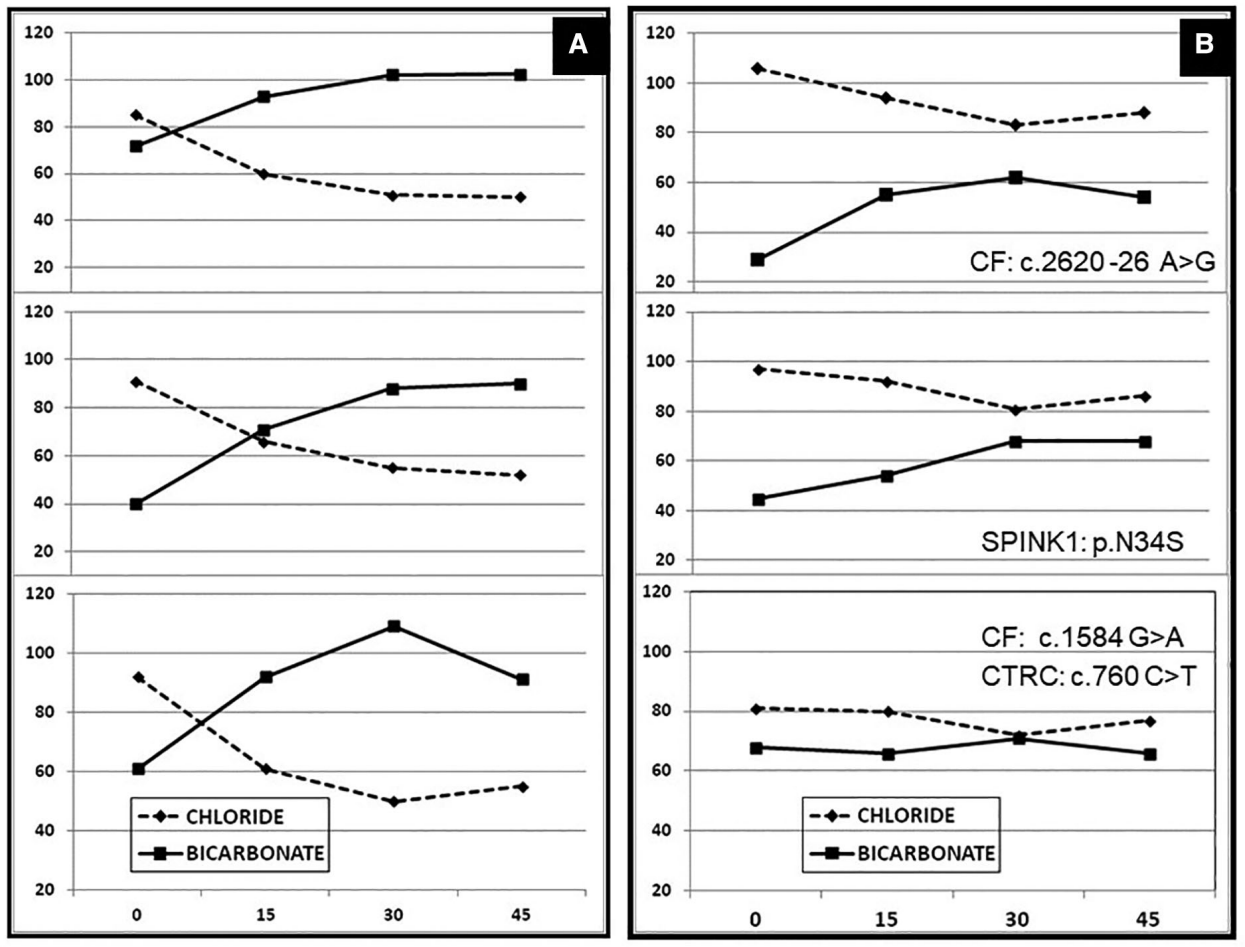
Ductal function assessment with bicarbonate concentration from prolonged ePFT with fluid collection up to 45 min. (**A**) Normal tests with the bicarbonate concentration is above 80 mmol/L. (**B**) Abnormal tests in patients with genetic abnormalities in three patients when the bicarbonate never reached the 80 mmol/L [adapted from Horvath, K. et al. (125)].

A method used at Arnold Palmer Hospital for Children, includes performing longer duration pancreatic fluid collection (45 min) after IV secretin administration in children where duct dysfunction was suspected. Those who had abnormal test result had genetic tests ordered. Figure 2 shows three cases with normal function and three abnormal test results with the genetic tests results added (125).

#### ePFT for ductal function in adults

After IV secretin administration, high bicarbonate secretion continues for a longer duration. Many adult studies used a 60 min collection time to assess ductal function and this subsequently led to longer anesthesia time.

A prospective ePFT study in patients (>16 years) with cystic fibrosis and healthy normal subjects administered the secretin 25 min before the endoscope insertion and collected the pancreatic fluid between 30 and 45 min (134) that significantly shorter than the 60 min test. The ePFT differentiated pancreatic-sufficient and insufficient patients with a sensitivity of 100% and specificity of 88%. Based on this study the 15 min collection was found to be sufficient to diagnose duct cell dysfunction. When CCK administration was added to secretin during ePFT it did not improve the accuracy of diagnosing EPI in adults with chronic pancreatitis (146). A similar conclusion was reported in pediatrics (135).

**Figure 3:**
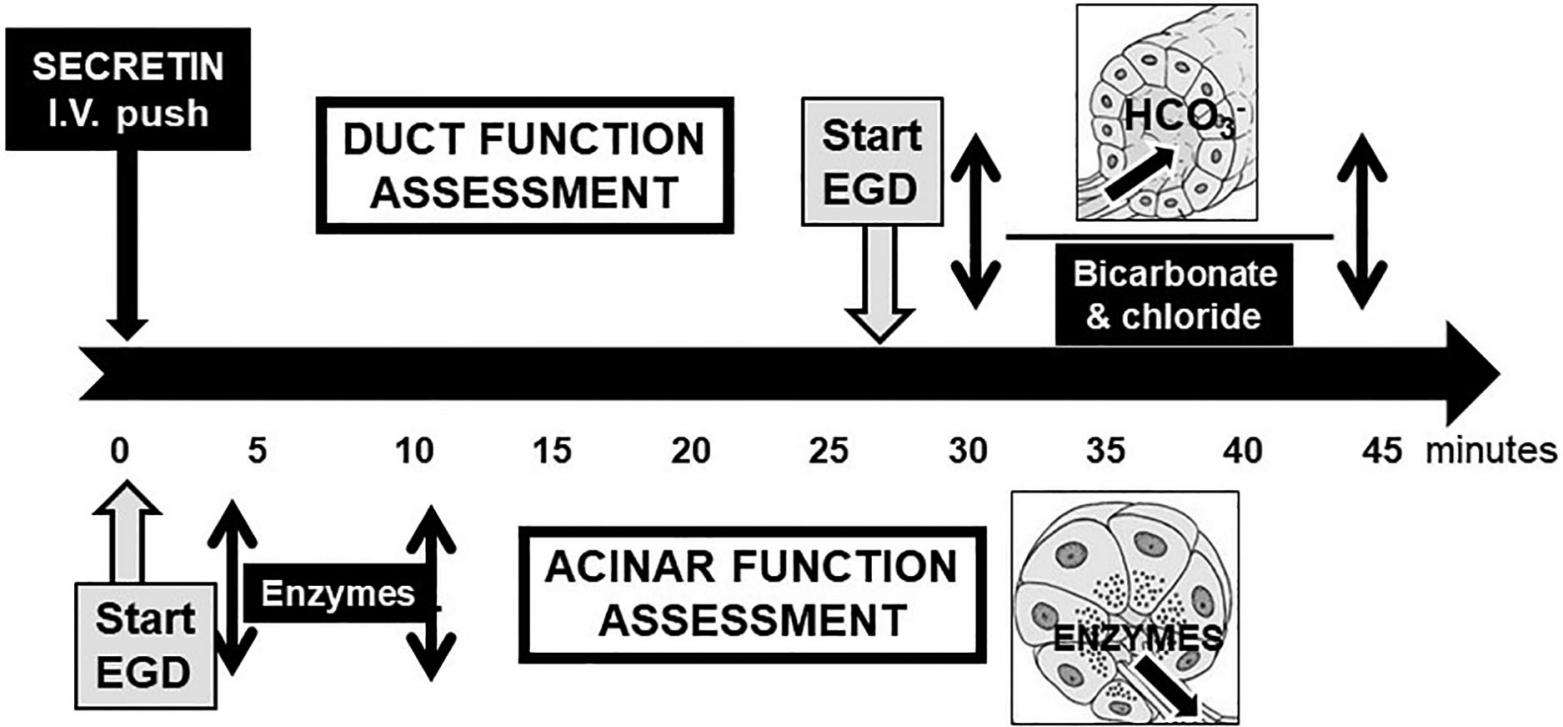
This flowchart shows the optimal time of fluid collection for acinar and ductal function after IV push administration of secretin [modified from Engjom, T. et al. (134)].

Figure 3 illustrates when the ePFT can be used for acinar and duct cell function assessment by using IV secretin administration.

## Imaging modalities to assess pancreatic exocrine function

Imaging studies are important in evaluation of anatomy of the pancreas as it relates to its function and thus should be utilized in assessing causes of exocrine pancreatic dysfunction. Of all imaging studies available, the secretin enhanced MRI (s-MRI) is the only one that can highlight functionality of the exocrine pancreas by evaluating fluid secretion.

Imaging studies can detect chronic pancreatitis typically when >50% of the gland is fibrotic (147). Thus, when it comes to assessing early stages of chronic pancreatitis in children with negative imaging studies, the combination of ePFT and endoscopic ultrasound should be considered (148). Additionally, identifying early stages of chronic pancreatitis utilizing these methods may also lead to improved outcomes with total pancreatectomy with autologous islet cell transplant (149).

### Ultrasound

Usually, ultrasound is the initial imaging modality in any suspected pancreatic disease as it can assess the size of pancreas, presence of peripancreatic fluid, the size and irregularity of the main duct and the presence of calcifications. Its sensitivity is 50% to 80% in adults (150).

### Magnetic resonance cholangio-pancreatograpy (MRCP)

It is the test of choice as it is more sensitive, does not require radiation and can image ducts as small as 1 mm (151) and enables to detect biliary stones and anatomical variants, such as pancreas divisum. Visualization of the pancreatic ducts are enhanced by the administration of secretin, which induces fluid secretion (152), this is the secretin-MRI (s-MRI).

The s-MRI is potentially a useful method to assess exocrine function by measuring the volume of the secreted fluid. Madzak et al*.* evaluated s-MRI in patients with CF and healthy patients (mean age 21 years) and found that CF patients with EPI had lower diffusion coefficient before secretin in the pancreatic head and lower secreted bowel fluid volumes (*P* = 0.035) (153). The s-MRI was also studied in pediatric population by Trout et al*.* who measured the secreted fluid in 50 healthy children and reported an association between the secreted volume and body surface area. They concluded that a secreted volume <43 ml or a secretion rate <2.3 ml/min (5th percentile values) can be considered abnormal in children (154).

### Endoscopic ultrasonography (EUS)

Endoscopic ultrasound involves the use of a specialized endoscopic device with ultrasound capability. Given that it is performed during endoscopy, it is considered an invasive technique and it is highly operator dependent (155). It provides highly accurate images of pancreatic ducts and parenchyma. When utilized in children, EUS can be both diagnostic and therapeutic. For example, imaging of the pancreas with EUS followed by an EUS-guided fine needle aspiration or biopsy can be useful in the diagnosis of idiopathic fibrosing pancreatitis or autoimmune pancreatitis (156). Additionally, microlithiasis can be identified by EUS as a possible contributor to acute recurrent pancreatitis in children (156). As a therapeutic modality, it can be used for the internal drainage of pancreatic pseudocysts as a complication of acute pancreatitis.

The role of EUS in evaluating the exocrine function of the pancreas was studied prospectively in 128 adult patients with EUS criteria of chronic pancreatitis and it was compared with the ^13^C-Mixed triglyceride breath test. They found that diagnosis of EPI increased linearly with the number of EUS criteria, and that the presence of intraductal calcifications, hyperechogenic foci with shadowing, and dilation of the main pancreatic duct were significantly and independently associated with EPI (157).

## Conclusions and future directions

In conclusion, accurate assessment of pancreatic function is essential in children with clinical concerns for maldigestion and malabsorption. EPI can be caused by several etiologies including developmental delays in enzyme maturation, isolated deficiencies, genetic disorders, and chronic pancreatitis. These can be easily missed as symptoms of EPI are often non-specific. Therefore, early diagnosis and treatment are important for improved outcomes in children.

Many studies showed that indirect measures of pancreatic function are unable to detect mild and moderate exocrine dysfunctions. Among the indirect non-stimulatory tests, FE-1 is the mostly used and most convenient test but its sensitivity and specificity is low compared with the direct function tests.

Although the Dreiling tube test was considered “the gold standard” for direct pancreatic function testing in the past, it is an unacceptable means of studying pancreatic exocrine function in children. ePFT is now the preferred method as it is technically easy to perform during upper gastrointestinal endoscopy, shorter in duration, and has comparable value with the Dreiling tube method.

The ePFT can be performed when routine endoscopy is obtained for investigation in children who are suspected of having malnutrition secondary to pancreatic exocrine dysfunction. It can detect both isolated and generalized deficiencies even if they are mild or moderate degree deficiencies. Like the “gold standard” Dreiling tube test collection, there is no uniformly accepted protocol for the ePFT. Although based on the pancreatic physiology fluid collection between 4 and 10 min reliable to assess the acinar cell function.

A multicenter study is needed for the standardization of ePFT in large number of children undergoing ePFT utilizing a single and uniform protocol.
